# *Coxiella burnetti* prosthetic joint infection in an immunocompromised woman: iterative surgeries, prolonged ofloxacin-rifampin treatment and complex reconstruction were needed for the cure

**DOI:** 10.1186/s42836-021-00097-1

**Published:** 2021-12-02

**Authors:** Patrick Miailhes, Anne Conrad, Chantal Sobas, Frederic Laurent, Sebastien Lustig, Tristan Ferry, on behalf of the Lyon BJI study group, Tristan Ferry, Florent Valour, Thomas Perpoint, Florence Ader, Sandrine Roux, Agathe Becker, Claire Triffault-Fillit, Anne Conrad, Cécile Pouderoux, Pierre Chauvelot, Paul Chabert, Johanna Lippman, Evelyne Braun, Sébastien Lustig, Elvire Servien, Cécile Batailler, Stanislas Gunst, Axel Schmidt, Elliot Sappey-Marinier, Quentin Ode, Michel-Henry Fessy, Anthony Viste, Jean-Luc Besse, Philippe Chaudier, Lucie Louboutin, Adrien Van Haecke, Marcelle Mercier, Vincent Belgaid, Aram Gazarian, Arnaud Walch, Antoine Bertani, Frédéric Rongieras, Sébastien Martres, Franck Trouillet, Cédric Barrey, Ali Mojallal, Sophie Brosset, Camille Hanriat, Hélène Person, Philippe Céruse, Carine Fuchsmann, Arnaud Gleizal, Frédéric Aubrun, Mikhail Dziadzko, Caroline Macabéo, Dana Patrascu, Frederic Laurent, Laetitia Beraud, Tiphaine Roussel-Gaillard, Céline Dupieux, Camille Kolenda, Jérôme Josse, Fabien Craighero, Loic Boussel, Jean-Baptiste Pialat, Isabelle Morelec, Michel Tod, Marie-Claude Gagnieu, Sylvain Goutelle, Eugénie Mabrut

**Affiliations:** 1 Service de Maladies Infectieuses et Tropicales, Hôpital de la Croix-Rousse, Hospices Civils de Lyon, 103 Grande-Rue de la Croix-Rousse, 69004 Lyon, France; 2 Centre Interrégional de Référence pour la prise en charge des Infections Ostéo-Articulaires complexes (CRIOAc Lyon), Lyon, France; 3 Université Claude Bernard Lyon 1, Lyon, France; 4 CIRI – Centre International de Recherche en Infectiologie, Inserm, U1111, Université́ Claude Bernard Lyon 1, CNRS, UMR5308, Ecole Normale Supérieure de Lyon, Lyon, France; 5 Laboratoire de Bactériologie, Institut des Agents Infectieux, Hôpital de la Croix-Rousse, Hospices Civils de Lyon, Lyon, France; 6 Service de Chirurgie Orthopédique, Hôpital de la Croix-Rousse, Hospices Civils de Lyon, Lyon, France

**Keywords:** Q fever, Prosthetic joint infection, Rheumatoid arthritis, Anti-TNF-α, *Coxiella burnetii*

## Abstract

**Background:**

Q fever is a zoonotic disease caused by the bacterium *Coxiella burnetii*, a strictly intracellular pathogen that can cause acute and chronic infection. Chronic Q fever can occur in immunocompetent as well as in immuno-compromised hosts, as a persistent localized infection. The main localizations are endocardial, vascular and, less frequently, osteoarticular. The most frequent osteoarticular form is spondyliscitis. Recommended treatment is combined doxycycline and hydroxychloroquine for 18 months, with cotrimoxazole as another option. *Coxiella burnetti* infection has been implicated in rare cases of prosthetic joint infection (PJI), and the medical and surgical management and outcome in such cases have been little reported.

**Case presentation:**

We report an unusual case of chronic Q fever involving a hip arthroplasty in an immunocompromised woman treated with tumor necrosis factor (TNF)-α blockers for rheumatoid arthritis. Numerous surgical procedures (explantation, “second look”, femoral resection and revision by megaprosthesis), modification of the immunosuppressant therapy and switch from doxycycline-hydroxychloroquine to prolonged ofloxacin-rifampin combination therapy were needed to achieve reconstruction and treat the PJI, with a follow-up of 7 years.

**Conclusions:**

*Coxiella burnetti* PJI is a complex infection that requires dedicated management in an experienced reference center. Combined use of ofloxacin-rifampin can be effective.

## Background

Q fever is a zoonotic disease caused by the bacterium *Coxiella burnetii*, a strict intracellular pathogen that could be responsible for acute and chronic infection [1–5]. Acute Q fever may symptomatic in only 40 % of cases and presented as an influenza-like illness, atypical pneumonitis, hepatitis and more rarely meningoencephalitis and myocarditis. Chronic Q fever could occur in immunocompetent as well as in immunocompromised hosts, and corresponds to persistent localized infection [1–5]. Main localizations are endocarditis, vascular infection, and, at a lesser extent, osteoarticular infections [5–18]. Among osteoarticular localizations, the most frequent clinical presentation is spondyliscitis [5]. Recommended treatment consists of the combination use of doxycycline and hydoxycloroquine, for 18 months, and cotrimoxazole should be considered as another option [1, 16]. *Coxiella burnetti* has been rarely identified as agent of prosthesis joint infection (PJI), and the medical and surgical management of such localization and the outcome, are poorly described [6, 16, 18, 19].

## Case presentation

In October 2013, a 65-year-old woman presented to our infectious disease center for a Q fever involving a hip arthroplasty. Her medical history revealed a hypothyroidism and a rheumatoid arthritis known since 1990, in remission since numerous years with a combination of methotrexate (15 mg/week) and tumor necrosis factor (TNF)-α blocker (adalimumab one infusion at 40 mg/month) therapy. Previously, she underwent hip arthroplasties, first in 2002 for the left side and then in 2003 for the right one.

**Fig. 1:**
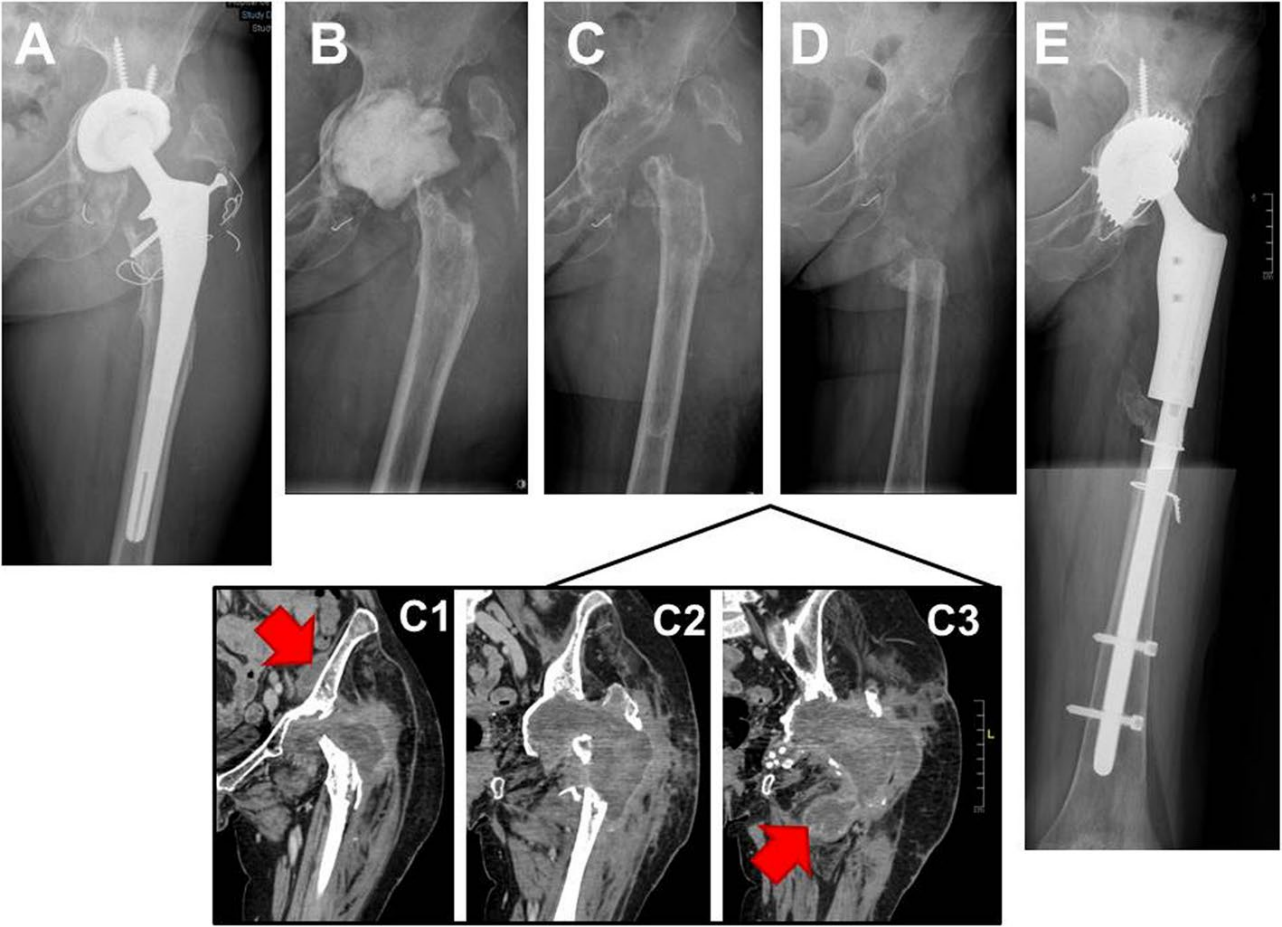
X-ray and CT-scan of the left hip performed: **A** at admission; **B** after prosthesis explantation, debridement and set-up of a spacer; **C** after performance of a new debridement and spacer removal, unfortunately with recurring abscesses visible on CT-scan (C1: ilio-psoas recurrent abscess, red arrow; C2: large collection within the joint; C3: abscesses in the thigh, red arrow) despite prolonged hydroxychloroquine-doxycycline treatment; **D** after a subsequent surgery with debridement of the recurrent abscesses and performance of a femoral resection; **E** 6 years after reimplantation of a megaprosthesis, no prosthesis loosening

**Fig. 2:**
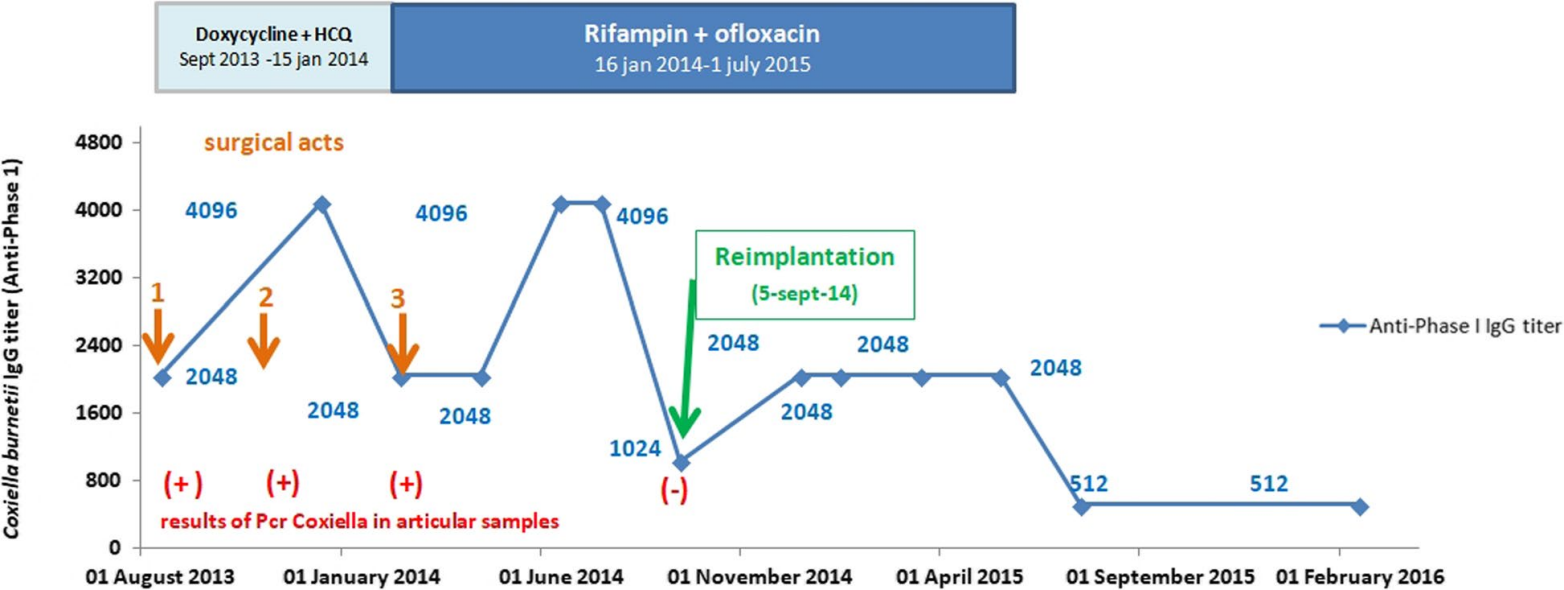
Kinetics of anti-phase I IgG titers (all previous sera were usually but not systematically analyzed in parallel) and results of the specific *coxiella* PCR during antimicrobial therapy and surgical procedures according to immunofluorescence assays. The Ct of the specific PCR (Smartcycler), when positive, was respectively 33.7, 31.3 and 24.6. Notes: HCQ = Hydroxychloroquine. Surgical acts: the first was performed on the 6th, August 2013, the second the 29th, October 2013 and the third, the 15th, January 2014

Three months ago, she was first admitted into her referential rheumatology center with a 1-month history of night fever and with a 2-week history of inflammatory pain of left hip. The fever appeared two days after a tooth extraction despite an oral antibiotic prophylaxis combining metronidazole and spiramycine. Erythrocyte sedimentation rate (ESR) and C-reactive protein (CRP) were elevated (76 mm/h and 55 mg/l, respectively). X-ray revealed loosening of the acetabular part of the prosthesis, and attachment of the greater trochanter (Fig. 1, panel A). Ultrasonography of the left hip revealed an intra-articular effusion and a large periprosthetic collection extending into the left iliac fossa. A first joint aspiration of the left hip revealed purulent synovial fluid, with 560 leukocytes/ml (52 % of monocytes, 39 % of lymphocytes and 9 % of neutrophils). Conventional blood and joint aspirate cultures including those of mycobacteria remained negative. Culture-negative PJI was suspected, adalimumab was discontinued (last injection in July 2013), and a resection hip arthroplasty was performed on the 6th, August 2013. All components were resected and a gentamicin-loaded spacer was implanted (Fig. 2, panel B). Operative findings showed abundant periprosthetic turbid fluid. Because of suspected dental origin, antimicrobial treatment combining ceftriaxone (1.5 g/12 h) and clindamycin (600 mg/8 h) was given in postoperative frame. Prolonged bacterial cultures (over a 14-day period) of five periprosthetic tissue samples remained sterile and histological findings revealed aspecific inflammation (irregular bone trabeculae with an inflammatory infiltrate composed by mononuclear and lymphohistiocytic cells, and also with few polymorphonuclear cells). For this culture-negative PJI, serological analysis was conducted in the bacteriology laboratory of Lyon Hospital (Immunofluorescence assay, Focus diagnostics, Cypress, USA) for Q fever and revealed significant titers (phase I; IgG 1:2048 [cut-off limit of positivity, 256] without IgM or IgA; phase II: IgG 1:512 without IgM or IgA) (Fig. 2). Also, both universal PCR (routinely performed for culture-negative PJI in our institution) and specific PCR for *Coxiella burnetii* (real-time PCR targeting the IS1111a insertion element [Progenie molecular provided by Orgentec SAS, Trappes France, [5]]) were performed from the first operative synovial sample (collected on the 6th of August 2013) and the result turned out to be positive. In early September, the first transthoracic echocardiogram was negative for endocarditis, and antimicrobial therapy was modified (oral doxycycline 200 mg/day and hydroxychloroquine 600 mg/day). In late September, because doxycycline trough plasma concentrations was low (1.8 mg/l), doxycycline was increased to 300 mg/day and concentrations in October was > 5 mg/l (9.7 mg/L). Hydroxychloroquine trough concentration (measured in October by high performance liquid chromatography-DAD) was 0.67 mg/L, which was considered as slightly low (the targeted level for trough concentration is 0.8–1.2 mg/L).

**Fig. 3:**
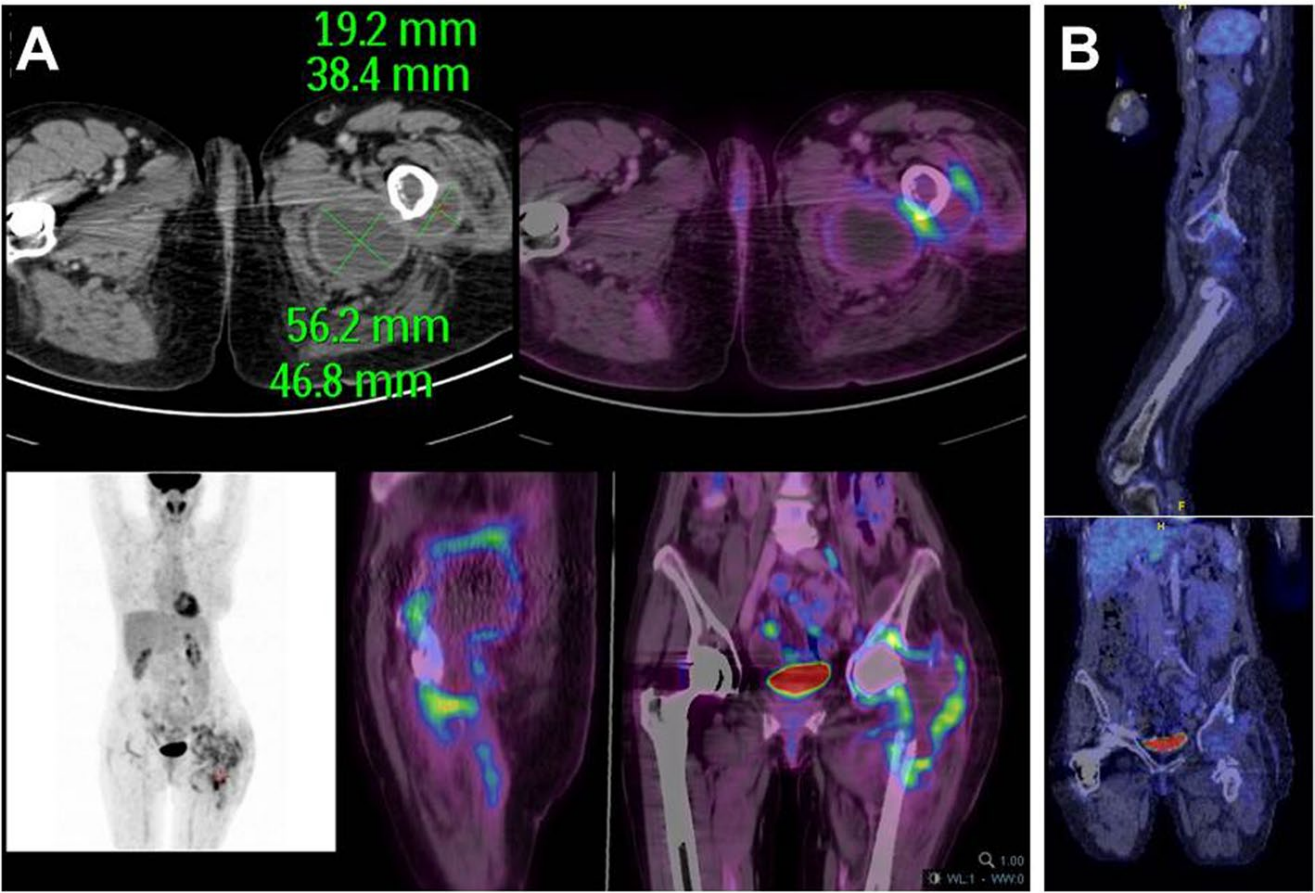
Positron emission tomography/computed tomography (PET-CT) performed ~ 3 months after the prosthesis explantation, showing a large ilio-psoas muscle collection (47 × 56 mm) communicating with numerous periprosthetic collections of the left hip (panel **A**). A second PET-CT was performed in June 2014, after the third surgical procedures and at ~ 6 month of rifampin and ofloxacin combination therapy, showing no persistent inflammatory process of the left hip and thigh (panel **B**)

At admission, in our regional reference center *Centre de Référence des Infections Ostéo-Articulaires complexes* (CRIOAc) Lyon (http://www.crioac-lyon.fr), she had no fever, no inflammatory scar nor left hip pain. Physical examination found a grade 3/6 mitral systolic murmur without sign of left ventricular insufficiency nor splenomegaly. Laboratory tests showed a normal leukocyte count, a mildly elevated CRP (22 mg/l) normal renal function and liver enzymes. Retrospectively, we learned that in the last days of April, she made a cruise in Mediterranean with one stopover in Morocco where she stayed near a cattle market. A transesophageal echocardiography (TEE) revealed mitral grade 2 regurgitation on a thickened valve without vegetation. A positron emission tomography/computed tomography (PET-CT) allowed the detection of a large ilio-psoas muscle collection (47 × 56 mm) communicating with numerous periprosthetic collections of the left hip (Fig. 3, panel A). Since the patient had received anti-TNF-α therapy, a mycobacterial infection needed to be ruled out and a second surgical procedure was performed, especially as the outcome seemed not to be favorable on combination use of doxycycline-hydroxychloroquine. A new debridement with flattening of three abscesses was realized and the spacer was removed (Fig. 1, panel C). As *C. burnetti* is not susceptible to gentamicin and, as a spacer is a foreign body facilitating the persistence of particular pathogens, we decided not to use another spacer. All the bacterial and mycobacterial specimens, realized during this second surgical time, remained negative, excluding the diagnosis of superinfection. Unfortunately, it was not feasible to try to culture *C. burnetii*, as fresh samples were needed to be sent to a reference lab, given thattime for surgery and time for sampling and for the shipment were not compatible. The specific PCR for *C. burnetii* from intra-articular sample was still positive. The diagnosis of persistent *C. burnetti* PJI was confirmed, despite explantation and adequate antimicrobial therapy. As a new debridement was performed with drainage of abscesses and removal of the spacer, and as the positive effect of doxycycline-hydroxychloroquine could take time, we maintained the doxycycline-hydroxychloroquine combination therapy.

Unfortunately, in mid-December, a CT scan showed the recurrence of large peri-articular abscesses (Fig. 1, panel C1-C3) as the same time as an elevation of inflammatory markers (CRP = 174 mg/l). Anti-phase I *C. burnetti* IgG titers remained high (Fig. 2). On January the 15th 2014, a third surgical debridement and irrigation was performed with new debridement of abscesses and resection of the proximal femur and the greater trochanter (Fig. 1, panel D). Again, *C burnetii* PCR was positive, both from the greater trochanter and muscle specimens, but without other bacterial or mycobacterial identification. Q fever serology remained positive with persistence of high level of anti-phase I IgG titer at 1:4096 (Fig. 2). Then, the doxycycline/hydrochloroquine combination was stopped, as it was considered as inefficient, and ofloxacin (400 mg/day) plus rifampin (900 mg/day) were introduced.

In February 2014, a second TEE ruled out endocarditis and a CT scan excluded aortic aneurysm. However, a peri-articular and two muscular collection(s) were still lasting, and were treated by a radiologic drainage with washing with mild efficacy. At the same time, with this particular chronic course, methotrexate given for rheumatoid arthritis was interrupted.

Three months after, she seemed better with no fever and all peri-articular hip collections slowly disappeared in CT scan imaging. However, in late April, an inflammatory flare of rheumatoid arthritis has led to again introduce methotrexate at 15 mg every week. In June 2014, a second PET-CT was performed (Fig. 3, panel B), showing no persistent inflammatory process of the left hip and thigh although anti-phase I IgG titer was flattening at 1:4096.

On September the 5th 2014, a left hip reimplantation was performed by using megaprosthesis. Both universal and specific PCR for *C. burnetii* were performed from bone tissue and remained negative. In January 2015, for better control of rheumatoid arthritis, methotrexate needed to be increased to 20 mg/week but without anti-TNF therapy because she had shingles one month earlier. Antimicrobial therapy with rifampin and ofloxacin was prolonged during 18 months after the last debridement and discontinued on the 1th, July 2015. At that time, Q fever serology titer dropped to 1:512 (that took time, > 18 months, since the last curative surgery and the switch to rifampin-ofloxacin antimicrobial treatment) and was unchanged at the last evaluation in February 2016 (Fig. 2). In July 2020, 7 years after the admission, the patient had no pain and no sign of PJI, had no prosthesis loosening detectable on X-ray (Fig. 1, panel E), and was still treated with 20 mg of methotrexate every week for rheumatoid arthritis.

To conclude, numerous surgical procedures (including explantation, “second look”, femoral resection), withdrawal of the TNF-α blocker and, then, of methotrexate, all being probably involved in the chronicity of the infection, switch from doxycycline-hydroxycholoquine to prolonged ofloxacine-rifampin combination therapy and finally reimplantation of a megaprosthesis after control of the infection were needed to achieve the cure of this relapsing chronic Q fever infection.

## Discussion and conclusions

*C. burnetti* is a zoonotic pathogen responsible for infection in *Bovidae* such as goats, sheep and cattle. During infection, it can induce premature birth. It is found in birth products (placenta), but also in urine, stool and milk. Human contamination occurs after inhalation of contaminated dust containing the bacterium. *C. burnetti* is an obligate intracellular pathogen that replicates in the host cell cytoplasm; consequently, it never grows in conventional cell-free bacterial culture. Q fever, which can be acute (such as pneumonia) or chronic (such as endocarditis or BJI), is the infectious disease caused by *C. burnetii*. It is treatable only by antibiotics that can penetrate host cells (such as doxycycline but not gentamicin), as *C. burnetti* is an obligate intracellular pathogen.

Chronic Q fever was rarely associated with an osteoarticular involvement, with only 1 % in large series reported by Raoult *et al* [4, 5]. Moreover, osteoarticular *C. burnetti* infections are largely heterogenous, and, unfortunately, few data are available about the medical and surgical management of such localizations [6–16, 18]. In pediatric cases, Q fever occurred often in healthy children, with a particular presentation of sequential osteomyelitis (4 out of 6) defined as chronic relapsing multifocal osteomyelitis [8, 11]. For adults’ cases, chronic Q fever may be presented as tenosynovitis, spondylodiscitis, osteomyelitis mainly of long bones and septic arthritis [5, 8, 14]. Few *C. burnetti* PJI have been described. Million *et al* reported that PET-CT could be used for the diagnosis of *C. burnetti* PJI and Melenotte *et* *al* proposed diagnostic criteria for this particular implant-associated bone and joint infection [5, 18]. Concerning the case presented here, positive PCR *C. burnetii* from a synovial sample is a definite criterion of *C. burnetii*-related PJI. Tande *et al* reported the favorable outcome of a conservative approach with performance of Debridement Antibiotic and Implant Retention (DAIR) procedure, followed by oral ciprofloxacin-doxycycline and then hydroxychloroquine-doxycycline combination. However, the patient was not immunosuppressed, there was no mention of prosthesis loosening, and the first clinical symptoms appeared only two months before the debridement [6]. Weisenberg *et al* reported a case that was managed with a 2-stage exchange and prolonged hydroxychloroquine-doxycycline combination [19]. Another case of *C. burnetti* PJI was reported by Meriglier *et al*, and the case developed prosthesis loosening and pseudarthrosis led to transfemoral amputation, and the patient received hydroxychloroquine-doxycycline for 18 months [16].

Here, we described a case of persistent *C. burnetti* PJI despite explantation, iterative debridements, and prolonged hydroxychloroquine-doxycycline therapy with optimal plasma concentrations. Bone resection and switch to ofloxacin-rifampin combination controlled the infectious process; allowed for the implantation of the megaprosthesis; and finally led to the cure. Both innate and adaptative immunity contributed to the control of Q fever. The immunosuppressive effects induced by methotrexate and anti-TNF therapy probably favored the chronicity of the Q fever in our patient. Methotrexate inhibits activation of nuclear factor-κB and inhibits important proinflammatory properties of major cell lineages involved in rheumatoid arthritis pathogenesis, such as T cells, macrophages, endothelial cells and fibroblast-like synoviocytes [20]. Secondly, TNF-α blokers may have delayed the clearance of *C. burnetii* in our patient, and probably facilitated the development of chronic Q fever, as it has been described by Schoffelen *et al* [21]. Mild CD4 lymphopenia has been associated with chronicity of *C. burnetii* infection in patients with endocarditis [22], and the role of T lymphocytes was confirmed because acute Q fever always evolved chronically in an athymic model of mice [23]. T cells response is critical for clearance of *C. burnetii*, as shown in mice models, particularly in nude mice and in IFN-γ knock-out mice [24]. Conversely, TNF-α-deficient mice had only a modest susceptibility to *C. burnetii* infection. However, Toll-like receptors (TLR)-2-deficient mice are highly permissive for *C. burnetti* infection by downregulating pro-inflammatory cytokines such as TNF-α [25]. Thus, in our case we could hypothesize that methotrexate and TNF-α blocker probably delayed the clearance of *C. burnetii*, and had enhanced the risk of chronic *Coxiella* infection in this immunodepressed woman. Also, the poor initial evolution despite adapted dose of doxycycline and hydroxychloroquine and two previous surgical procedures may be explained, at least in part, by the continuation of methotrexate in our case.

Interestingly, this chronic osteoarticular Q fever was characterized, as in most children cases, by a multifocal relapsing clinical course necessitating three surgical procedures (the explantation of prosthesis and two surgical debridements with irrigation of abscesses and resection of greater trochanter). In our case, the PET-CT imaging helped us to define the time of prosthesis reimplantation.

Q fever diagnosis is usually based on serological assays, and chronic stage is associated with anti-phase I IgG titer above 1:800 [26]. As some recent studies reported, specific *C. burnetii* PCR assay of tissue samples may be a useful tool to early establish a diagnosis of Q fever [8, 11]. A negative PCR result for *C. burnetii* after a first positive test, as noted in our case, may also  suggest a good outcome under antimicrobial therapy. Indeed, in osteoarticular involvement, serological status changes may be different from that defined in Q fever endocarditis and in some cases serological titers may be on a plateau phase without clear failure or rapid relapse of Q fever after antibiotics interruption [11, 14].

Doxycycline combined with hydroxychloroquine is the mainstay of medical therapy for Q fever endocarditis [2, 3]. For osteoarticular chronic Q fever, in particular with PJI involvement, the best antimicrobial therapy is still not well-defined and methods of monitoring remain uncertain. Here we observed the reccurrence of clinical symptoms and abscesses under hydroxychloroquine-doxycycline treatment. Susceptibility testing of *C. burnetti* is not feasible in clinical practice, as it is a strict intracelullar pathogen, and it was not possible here to demonstrate doxycycline resistance *in vitro* [27–29]. Alternative antimicrobial regimens combining both good bone penetration, intracellular activity and activity in biofilm, such as rifampin and fluoroquinolone, may be a good choice, particularly in chronic osteoarticular infections or PJI. The combination of fluoroquinolone-rifampin is the cornerstone of the treatment of staphylococcal PJI, and to the best of our knowledge, there is no description of its use in *C. burnetti* PJI until now, whereas *C. burnetti* is usually considered to be sensitive to these antibiotics [6, 30–33]. Finally, as reported in *C. burnetii* infection of aortic aneurysms or vascular grafts [34], surgical procedure seems of major importance, in particular in patients with *C. burnetti* PJI, to achieve a healing.

*C. burnetti* PJI is infrequent, but probably underestimated. It may be suspected in case of negative bacterial and mycobacterial culture and also in culture-negative PJI. Both serological testing and specific PCR assay of tissue specimens are needed for prompt early diagnosis. Multidisciplinary management in a reference center (such as a CRIOAc in France) that includes orthopedists, infectiologists, bacteriologists and radiologists is essential to obtaining a chance of a cure [35]. Iterative surgeries may be required, and clinically effective anti-infection drugs are mandatory before performing reconstruction, which is usually complex in the case of bone resection. As *C. burnetti* can persist under hydroxychloroquine-doxycycline treatment, prolonged combined ofloxacin-rifampin could be an option.

## Data Availability

Not applicable.

## Abbreviations

PJI: Prosthetic joint infection
TNF-α: Tumor necrosis factor-α
ESR: Erythrocyte sedimentation rate
CRP: C-reactive protein
PCR: Polymerase chain reaction
CRIOAc Lyon: Centre de Référence des Infections Ostéo-Articulaires complexes
TEE: Trans-esophageal echocardiography
PET-CT: Positron emission tomography/computed tomography
CT scan: Computed tomography scan
DAIR: Debridement antibiotic and implant retention

## References

[1] Anderson A, Bijlmer H, Fournier P-E, Graves S, Hartzell J, Kersh GJ (2013). Diagnosis and management of Q fever–United States, 2013: recommendations from CDC and the Q Fever Working Group. MMWR Recomm Rep.

[2] Maurin M, Raoult D (1999). Q fever. Clin Microbiol Rev.

[3] Parker NR, Barralet JH, Bell AM (2006). Q fever. Lancet.

[4] Raoult D, Tissot-Dupont H, Foucault C, Gouvernet J, Fournier PE, Bernit E (2000). Q fever 1985–1998. Clinical and epidemiologic features of 1,383 infections. Medicine (Baltimore)..

[5] Melenotte C, Protopopescu C, Million M, Edouard S, Carrieri MP, Eldin C (2018). Clinical Features and Complications of Coxiella burnetii Infections From the French National Reference Center for Q Fever. JAMA Netw Open.

[6] Tande AJ, Osmon DR, Greenwood-Quaintance KE, Mabry TM, Hanssen AD, Patel R (2014). Clinical characteristics and outcomes of prosthetic joint infection caused by small colony variant staphylococci. mBio.

[7] Raoult D, Bollini G, Gallais H (1989). Osteoarticular infection due to Coxiella burnetii. J Infect Dis.

[8] Landais C, Fenollar F, Constantin A, Cazorla C, Guilyardi C, Lepidi H (2007). Q fever osteoarticular infection: four new cases and a review of the literature. Eur J Clin Microbiol Infect Dis.

[9] Maltezou HC, Raoult D (2002). Q fever in children. Lancet Infect Dis.

[10] Cottalorda J, Jouve JL, Bollini G, Touzet P, Poujol A, Kelberine F (1995). Osteoarticular infection due to Coxiella burnetii in children. J Pediatr Orthop B.

[11] Nourse C, Allworth A, Jones A, Horvath R, McCormack J, Bartlett J (2004). Three cases of Q fever osteomyelitis in children and a review of the literature. Clin Infect Dis.

[12] Garron E, Viehweger E, Launay F, Guillaume JM, Jouve JL, Bollini G (2002). Nontuberculous spondylodiscitis in children. J Pediatr Orthop.

[13] Ellis ME, Smith CC, Moffat MA (1983). Chronic or fatal Q-fever infection: a review of 16 patients seen in North-East Scotland (1967-80). Q J Med.

[14] Merhej V, Tattevin P, Revest M, Le Touvet B, Raoult D (2012). Q fever osteomyelitis: a case report and literature review. Comp Immunol Microbiol Infect Dis.

[15] Acquacalda E, Montaudie H, Laffont C, Fournier P-E, Pulcini C (2011). A case of multifocal chronic Q fever osteomyelitis. Infection.

[16] Meriglier E, Sunder A, Elsendoorn A, Canoui E, Rammaert B, Million M (2018). Osteoarticular manifestations of Q fever: a case series and literature review. Clin Microbiol Infect.

[17] Angelakis E, Edouard S, Lafranchi M-A, Pham T, Lafforgue P, Raoult D (2014). Emergence of Q fever arthritis in France. J Clin Microbiol.

[18] Million M, Bellevegue L, Labussiere A-S, Dekel M, Ferry T, Deroche P (2014). Culture-negative prosthetic joint arthritis related to Coxiella burnetii. Am J Med.

[19] Weisenberg S, Perlada D, Peatman T. Q fever prosthetic joint infection. BMJ Case Rep. 2017;2017. 10.1136/bcr-2017-220541 PMC5623254 28739619

[20] Cronstein BN, Aune TM (2020). Methotrexate and its mechanisms of action in inflammatory arthritis. Nat Rev Rheumatol.

[21] Schoffelen T, den Broeder AA, Nabuurs-Franssen M, van Deuren M, Sprong T (2014). Acute and probable chronic Q fever during anti-TNFα and anti B-cell immunotherapy: a case report. BMC Infect Dis..

[22] Sabatier F, Dignat-George F, Mège JL, Brunet C, Raoult D, Sampol J (1997). CD4 + T-cell lymphopenia in Q fever endocarditis. Clin Diagn Lab Immunol.

[23] Kishimoto RA, Rozmiarek H, Larson EW (1978). Experimental Q fever infection in congenitally athymic nude mice. Infect Immun.

[24] Andoh M, Zhang G, Russell-Lodrigue KE, Shive HR, Weeks BR, Samuel JE (2007). T cells are essential for bacterial clearance, and gamma interferon, tumor necrosis factor alpha, and B cells are crucial for disease development in Coxiella burnetii infection in mice. Infect Immun.

[25] Zamboni DS, Campos MA, Torrecilhas ACT, Kiss K, Samuel JE, Golenbock DT (2004). Stimulation of toll-like receptor 2 by Coxiella burnetii is required for macrophage production of pro-inflammatory cytokines and resistance to infection. J Biol Chem.

[26] Raoult D, Marrie T (1995). Q fever. Clin Infect Dis.

[27] Brennan RE, Samuel JE (2003). Evaluation of Coxiella burnetii antibiotic susceptibilities by real-time PCR assay. J Clin Microbiol.

[28] Spyridaki I, Psaroulaki A, Kokkinakis E, Gikas A, Tselentis Y (2002). Mechanisms of resistance to fluoroquinolones in Coxiella burnetii. J Antimicrob Chemother.

[29] Spyridaki I, Psaroulaki A, Vranakis I, Tselentis Y, Gikas A (2009). Bacteriostatic and bactericidal activities of tigecycline against Coxiella burnetii and comparison with those of six other antibiotics. Antimicrob Agents Chemother.

[30] Drancourt M, Stein A, Argenson JN, Zannier A, Curvale G, Raoult D (1993). Oral rifampin plus ofloxacin for treatment of Staphylococcus-infected orthopedic implants. Antimicrob Agents Chemother.

[31] Senneville E, Joulie D, Legout L, Valette M, Dezèque H, Beltrand E (2011). Outcome and predictors of treatment failure in total hip/knee prosthetic joint infections due to Staphylococcus aureus. Clin Infect Dis.

[32] Lora-Tamayo J, Murillo O, Iribarren JA, Soriano A, Sánchez-Somolinos M, Baraia-Etxaburu JM (2013). A large multicenter study of methicillin-susceptible and methicillin-resistant Staphylococcus aureus prosthetic joint infections managed with implant retention. Clin Infect Dis.

[33] Osmon DR, Berbari EF, Berendt AR, Lew D, Zimmerli W, Steckelberg JM (2013). Diagnosis and management of prosthetic joint infection: clinical practice guidelines by the Infectious Diseases Society of America. Clin Infect Dis.

[34] Botelho-Nevers E, Fournier P-E, Richet H, Fenollar F, Lepidi H, Foucault C (2007). Coxiella burnetii infection of aortic aneurysms or vascular grafts: report of 30 new cases and evaluation of outcome. Eur J Clin Microbiol Infect Dis.

[35] Ferry T, Seng P, Mainard D, Jenny J-Y, Laurent F, Senneville E (2019). The CRIOAc healthcare network in France: a nationwide Health Ministry program to improve the management of bone and joint infection. Orthop Traumatol Surg Res.

